# Development of a Piezo-Driven Liquid Jet Dispenser with Hinge-Lever Amplification Mechanism

**DOI:** 10.3390/mi11020117

**Published:** 2020-01-21

**Authors:** Mojiz Abbas Trimzi, Young Bog Ham, Byeung Cheol An, Young Min Choi, Jung Ho Park, So Nam Yun

**Affiliations:** 1 Department of Thermal Systems, Korea Institute of Machinery and Materials, Daejeon 34103, Korea or engrmojizabbas@hotmail.com (M.A.T.); abcn@kimm.re.kr (B.C.A.); anaud007@kimm.re.kr (Y.M.C.); jhpark@kimm.re.kr (J.H.P.); ysn688@kimm.re.kr (S.N.Y.); 2 Department of Plant System & Machinery, University of Science & Technology, Daejeon 34113, Korea

**Keywords:** droplet, jetting, needle-type, normally-closed, hinge-lever, dispenser, displacement amplification, nozzle

## Abstract

Owing to the quick response, compact structure, high precision, huge blocking force generation, and ease of operation, piezoelectric actuators are urgently being adopted in the field of advanced dispensing for jetting performance improvement and fulfillment of precision requirements in microelectronics packaging, adhesive bonding, and miniaturization industry. This research focuses on the fundamental design and development of a piezo-electrically driven compact fluid dispenser using the principle of a class-one lever for amplification of needle displacement, and enhancement of application areas of the developed jet dispenser. Using fundamental lever principle, geometry-based modelling is carried out to fabricate a working prototype of a normally closed hinge-lever type dispenser. Preliminary experiments are carried out to witness the workability of the fabricated dispenser to deliver 100 dots of working fluid per second that will provide a novel device for dispensing of various fluids, and the proposed amplification mechanism suits various other piezoelectric applications as well.

## 1. Introduction

The applications of liquid dispensing technology have abruptly increased in the past few decades because of the adoption of fluid dispensing in photovoltaic solar power industry, bio-medical devices, electronic packaging equipment, light-emitting diode (LED) encapsulation, and bonding or filling of non-electrical components [1,2,3,4,5,6,7,8]. In most of the circumstances, dispensers are required to generate accurate sized, high speed, and compact drops on demand thus the innovation in dispenser design has become a prerequisite to keep up with the demands of industry [3,9]. 

For the ease of understanding on broad-spectrum, dispensers can generally be categorized into contact and non-contact types [10,11]. The contact type dispensers with large driving force are widely applied for viscous adhesive jetting but the damage of the device is possible because the dispensing nozzle is kept closer to the substrate that results in low precision and risk of liquid contact pollution, which is undesired in the field of microelectronic packaging. They have low efficiency because the dispenser generates droplets by air pressure, piston or screw movement, thus the droplet size is hard to control and challenging for micro-device packaging in micro electro-mechanical systems (MEMS) and other areas of application [12,13,14,15,16]. Contrarily, the non-contact dispensers generate the droplets directly by instantaneous pressure gradient without the vertical motion of the substrate [11]. They have wide areas of application in microelectronic packaging because of the compact structure, high accuracy, and small droplet size which offers improved working efficiency by protecting the needle or tappet of dispenser eliminating the risk of device damage because of contact [11,12]. 

The piezoelectric actuators have numerous applications in the areas of needle-free drug delivery, vibration suppression, active shape control, energy harvesting, inkjet printing, and fuel injectors [17,18,19,20,21,22]. However, piezo actuators have one major drawback that is their small output displacements but because of their compact structure and easy usability, their demand and applications are increasing [23]. However, for a large number of applications, more displacement is required, so scientists came up with the ideas for the displacement amplification mechanisms to enhance their applications in the fields where larger displacements are fundamentally important. If necessary, the scope for applications of piezoelectric actuators is broaden by using displacement amplification mechanisms to enhance the stroke length of piezostack actuator, which include lever-type, moonie-type, rainbow-type, cymbal-type, ellipse-type, rhombus-type, bridge-type, rhombus with hinges, compound bridge-type, and three-dimensional displacement amplification mechanisms [17,24,25,26,27,28,29]. But the displacement amplification comes at the cost of blocking force reduction because of the displacement amplification structures [30]. One of the most commonly adopted displacement amplification structure is a rhombus-type compliant mechanism [31,32]. Its most of the aspects including enhanced mathematical modelling to simplified structure models and various application case studies with detailed experiments have been discussed since the past fifteen years [4,7,11,18,21,30,33,34,35,36]. The second famous displacement amplification in literature is a bi-piezoelectrically driven lever-type displacement amplification mechanism that is more recent and has been the focus of attention until now [8,9,12,37]. Similarly, some mechanisms are less famous that employ various kinds of piezo-actuators in novel configurations, either for dispenser fabrication or for the high-velocity jet injection [2,14,18,38]. In addition, amplified piezo-actuators are being used effectively in similar areas of applications as MEMS and in EHD inkjet technology, where similar mechanisms and approaches are used however properties of the working fluid and number of nozzles are the major priorities in such kind of system designs [22,39,40,41,42,43]. In short, the trend has been more leaning toward the amplified displacements in the past, but the research on parameters influencing the dispensing quality and the methods to improve the dispensing systems is being carried out these days. In this regard, authors want to contribute with the proposal of a simple and compact, piezo-driven, hinge-lever type dispenser that can be used for wide viscosity range of liquid dispensing.

This study mainly focuses on the fundamental design of a hinge-lever type jet dispenser driven by a piezoelectric stacked actuator [24]. The basic design model is proposed and geometry-based modelling of a proposed jet dispenser is carried out using SimulationX software by ESI ITI GmbH to observe the feasibility of design [44]. The principle of the class-one lever is adopted with a single nozzle and single-stacked piezoelectric actuator. The amplification ratio of the lever is selected so that the generated force on tappet is well enough to push a wide range of liquids with varying viscosities to enhance the areas of application of developed hinge-lever type jet dispenser. Moreover, the preliminary experimental setup is adopted for the measurement of output-amplified displacement of the tappet. Finally, the experiment for jetting of hundred liquid droplets per second is successfully witnessed using two liquids of different physical properties in addition to the exploration of newly developed jet dispenser workability on varying operating parameters and the dispenser has shown a satisfactory preliminary response. It is to be noted that the structural configuration of the piezo-driven jetting dispenser designed and manufactured in this study is essentially novel and a newly developed one which has not been reported and published by our group previously.

## 2. Motivation for Research

The best example of a class-one lever is seesaw that is a long narrow board supported by a single pivot point. When one end goes up, the other goes down. The core idea of current research is adopted from class-one lever and we have tried to implement it in the field of jet dispensing technology. 

**Figure 1:**
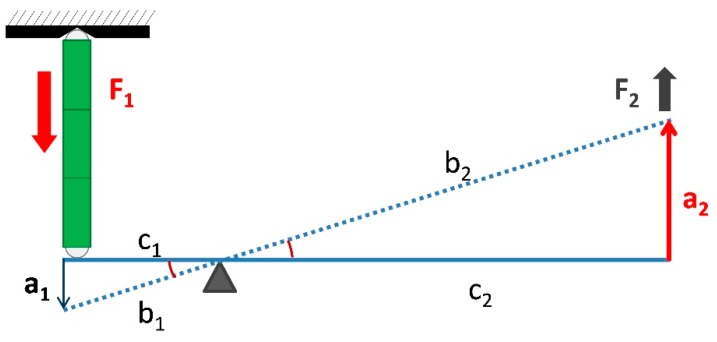
Schematic representation of the core mechanism for the class-one lever.

Figure 1 represents the schematic arrangement of the conceptual depiction for the dispenser. The piezoelectric actuator used in the dispenser development is a triplet piezostack, a commercial product of piezomechanik (type osi-stack PSt 150/7 × 7/60) [45]. The lever length *c*_1_ (1.5 mm) and *c*_2_ (9.5 mm) is defined, maximum blocking force *F*_1_ (3500 N) and maximum stroke *a*_1_ (60 μm) is taken from product catalogue and using Pythagoras theorem and class-one lever principle, as described in Equations (1) and (2), the output stroke *a*_2_ and output force *F*_2_ is calculated in addition to amplification ratio R. 

From Pythagoras theorem:

(1) $$a_{2}=\frac{a_{1}}{c_{1}}\times c_{2}$$

In Equation (1), *a*_2_ is the theoretical amplified output displacement of the needle that is responsible for jet dispensing.

From class-one lever principle:

(2) $$F_{2}=\frac{c_{1}}{c_{1}}\times F_{1}$$

Equation (2) represents the output force *F*_2_ at the long end on lever *c*_2_ that is resulted after the amplification of needle displacement. The output force is less than inlet force because of increased length *c*_2_ of the lever.

The simplified representation of the class-one lever provides us with useful results in terms of output displacement, amplification ratio, and resulting output force that are tabulated in Table 1.

## 3. Needle-Type Dispenser Simulation and Modelling

### 3.1. Dispensing System Overview and Working Principle

**Figure 2:**
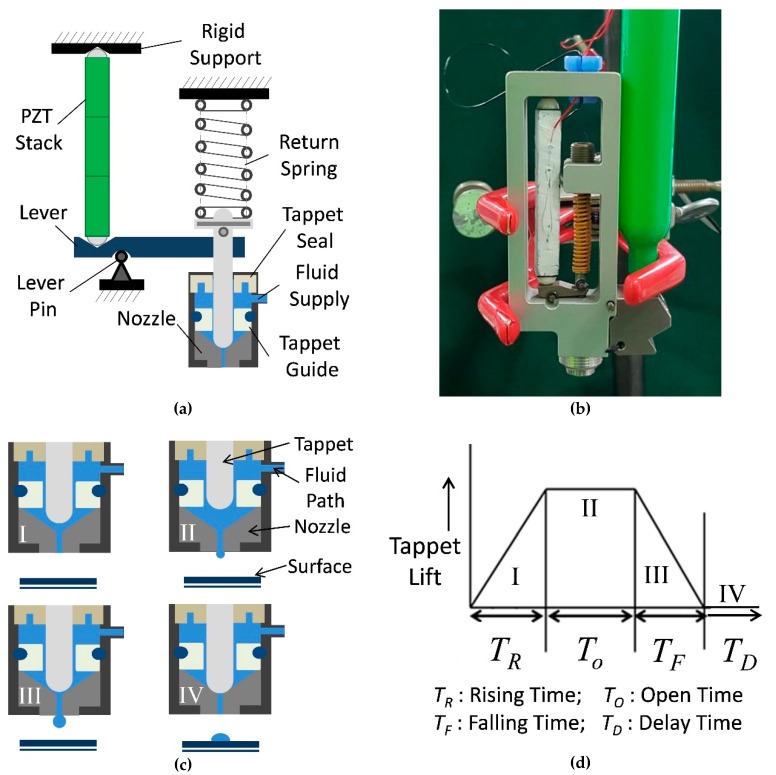
Configuration of the needle-type piezo-driven liquid jet dispenser and its dispensing mechanism (**a**) jet dispenser model with labelled components; (**b**) fabricated piezo-driven jet dispenser with a liquid syringe; (**c**) needle position with respect to dispenser driving cycle; (**d**) driving cycle for the jet dispenser.

In order to fabricate the working prototype of needle-type piezoelectric jet dispenser, a dispenser model is developed as presented in Figure 2a (see video S1 in Supplementary Materials). Figure 2a also represents the displacement amplification mechanism and jetting mechanism. Figure 2b presents the working prototype of a hinge-lever type piezo-driven dispenser. As shown in Figure 2a,b, one end of the triplet piezostack is attached and is fixed to PST adjustment nut inside a rigid metallic frame and this end of piezostack is rigid end. 

The piezostack actuator has ceramic hemispheres on both ends that are symmetrically distributed by strong bonding at both ends of the piezoelectric actuator to keep the blocking force equally distributed all over the piezostack as the unequal distribution of load over any end of piezostack may result in burning of piezostack because of short circuit or unequal stress distribution. The one end of the piezostack is placed over the short end of the lever so that the piezostack and short end of the lever form mechanical contact. The jetting mechanism is separated from the displacement amplification mechanism using a pivoting point named as lever pin or hinge. The jetting part of the jet dispenser consists of a tappet, tappet guide, nozzle, nozzle fixing bolt, liquid supply block with a fluid channel and temperature controller, spring, spring compression adjustment nut, frame adjusting nut, and liquid seal and bushing, as well as a tappet seal. The Luer fitter can be connected to the supply block that can be connected to the liquid containing syringe [46]. A problem that may arise during the assembly of the dispenser is the movement of lever-pin adopted to balance the piezostak and spring, and it can be solved by using small size snap-rings on each side of the lever pin to prevent lever-pin slip. Because of tension in the spring, the needle remains in contact with the end of the nozzle so that there is no leakage from the nozzle when the dispenser is closed. When the piezostack actuator is excited because of the applied voltage during dispensing, the long end of lever reciprocates inducing vertical movement in the tappet. Figure 2c,d shows a clear depiction of the jetting process during a complete driving cycle of the dispenser. When the voltage is applied to piezostack, the long lever arm is moved up, inducing further compression in return spring, thus driving the needle until it reaches the highest position. The whole dispensing process is divided into four stages namely stage I, II, III, and IV. In stage I, the piezostack is excited and the needle rises. At this point, fluid enters the gap between the nozzle and the tappet under feed pressure that is set according to working fluid properties and nozzle size. During stage II, the tappet reaches its maxim highest position against the spring force and the spring gets further compressed. In stage III, the piezostack actuator de-energizes and returns to its neutral state and the needle drops rapidly because of the compression in spring, producing a large shear force that overcomes the viscous forces of the working fluid, breaking the jet. Ultimately, at stage IV, the nozzle is closed by the tappet resting on its opening, and the fluid forms a droplet on the surface under the nozzle. All these stages (I–IV) make a full cycle of dispensing, with durations *T_R_*, *T_O_*, *T_F_*, and *T_D_* respectively. Therefore, a complete dispensing cycle can be named as *T*, whereas *T = T_R_ + T_O_ + T_F_ + T_D_* [37].

### 3.2. Governing Equations for Jet Dispenser Modelling

This section deals with the geometry-based modelling and simulation of hinge-lever type jet dispenser. The modelling and simulation are carried out using a professional version of SimulationX 3.9 software of ESI ITI GmbH, Germany [44]. The following assumptions are considered for simulation of dispenser system. (a) The fluid is incompressible and the flow of the fluid during jet formation does not deviate from the equation of continuity. In other words, the mass of the fluid flowing into any section should be equal to the mass of the fluid flowing out of any other section at the given time. (b) The effect of gravity is negligible because of the small flow channels. (c) The effect of shear force on fluid viscosity is negligible. (d) The fluid wall has no deformation and no-slip boundary conditions are considered. (e) The axial flow velocity is much higher than the radial flow velocity so that the radial flow is negligible.

Governing equations that are mainly used in fluid dynamics and hydraulics are employed for modeling of the dispenser system. Equation (3) represents the force balance acting on the object or inside the chamber under the hydraulic pressure, damping force and spring force. Equation (4) represents the relationship between the flow rate and pressure when fluid is passing through a flow resistance such as an orifice. Equation (5) depicts the Hagen-Poiseuille equation that refers to the flow rate caused by the pressure difference when the fluid is passing through a narrow and long cylindrical flow path. Equation (6) shows the pressure build-up because of the fluid flow inside and out of the chamber and changes in the volume of the chamber.

(3) $$F\left(t\right)=C_{1}x^{\prime \prime }+C_{2}x^{\prime }+C_{3}x$$

(4) $$Q=C_{d}A\sqrt{2∆P/\rho }$$

(5) $$Q=\;\frac{\pi r^{2}\left(∆P\right)}{8\mu L}$$

(6) $$\frac{dP}{dt}=\frac{\beta }{V_{o}}\;\sum \left(Q+\frac{dV}{dt}\right)$$

In addition, these equations are nonlinear as pressure is required to calculate the flow rate and the flow rate is required to calculate the pressure. A linear multi-step method is introduced to solve the nonlinear first-order ordinary differential equation and a fifth-order backward differentiation formula (5th BDF) as shown in Equation (7) is employed in the linear multi-step method [20].

(7) $$y_{n+6}-\frac{360}{147}y_{n+5}-\frac{450}{147}y_{n+4}-\frac{400}{147}y_{n+3}+\frac{225}{147}y_{n+2}-\frac{72}{147}y_{n+1}+\frac{10}{147}y_{n}=\frac{60}{147}hf\left(t_{n+6},y_{n+6}\right)$$

After linearization, calculations are analyzed by utilizing sparse matrix solver for result generation. The minimum step size is kept 0.1 ns and the minimum output step size is kept to be 0.1 μs. Flow coefficient Cx is used with its default value and SAE 15W-40 mineral oil is chosen as the working fluid in the dispenser model. Afterward, fluid properties such as density and bulk modulus are defined. The temperature is kept at 0 °C as a constant throughout the simulation. It is because the dynamic viscosity of SAE 15W-40 mineral oil at 0 °C is 1328.0 mPa·s, which is closely related to the viscosity of glycerin that is used for follow-up experiments that has a viscosity of 1412.0 mPa·s. In addition, the tappet is assumed to represent the ideal behavior according to the signal. The constant pneumatic pressure is simulated in the dispenser model with a supply of 0.2 MPa throughout the simulation [44]. 

### 3.3. Geometry Based Modelling of Dispenser

**Figure 3:**
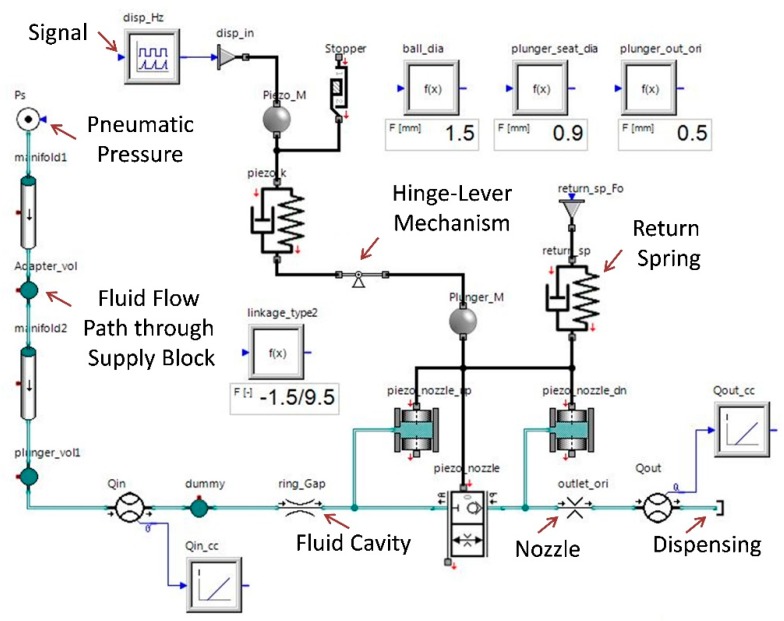
Geometry based modelling of the jet dispenser with a detailed simulation model representation of jet dispenser on SimulationX.

Figure 3 represents the detailed simulation model of the hinge-lever type jet dispenser. The dimensions of fluid flow channels are substituted in the model for simulation of the jetting phenomena. Each component of the dispenser is modelled with attention to detail. The amplification ratio is defined using hinge-lever mechanism from the mechanics library of the SimulationX software. A continuous pressure supply of 0.2 MPa of air pressure is modelled through the pneumatic library of the software. Similarly, manifolds, volumes, piezo-nozzle, and ring-gap components depict the flow channels of the liquid flowing in dispenser that are taken from the hydraulic library of the SimulationX software. In addition, piezostack and return spring in addition with tappet and nozzle components are simulated using the mechanics library from the software. Finally, a complete model of jet dispenser is simulated. The simulation provides results about the influence of input piezostack displacement and output needle or tappet displacement. The response characteristics of the hinge-lever mechanism, return spring behavior, and output-dispensed volume with respect to pressure gradient are some of the useful results obtained through this simulation. Table 2 represents crucial parameters utilized in the following simulation.

**Figure 4:**
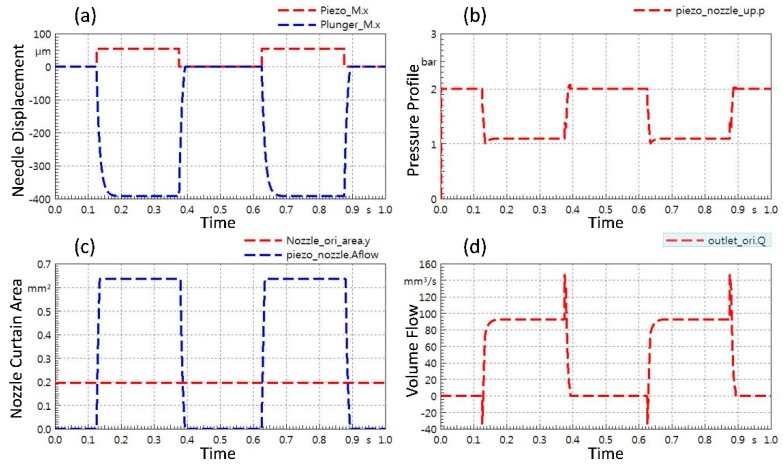
Simulation results for jet dispenser: (**a**) Plot of piezostack displacement and needle displacement; (**b**) pressure fluctuation with respect to tappet movement; (**c**) nozzle curtain area with reference of nozzle cross sectional area; (**d**) liquid volume flow through nozzle with respect to needle displacement.

Figure 4 represents the simulation results of the jet dispenser. The Figure 4a represents the input piezoelectric displacement because of the excitation as a red dotted line in micrometers which is 55–60 μm whereas the blue dotted line represents the response of needle (named plunger in the simulation). It can be observed that input displacement of piezoelectric actuator is positive whereas output amplified displacement of the dispenser needle is negative. The continuous supply of 0.2 MPa pressure is applied in simulation. Figure 4b portrays the overall pressure fluctuation profile with respect to the tappet movement. The cross-sectional area of nozzle outlet stays constant because it is fixed because of the nozzle geometry but the curtain area of nozzle increases and decreases because of the tappet displacement, which results in dispensing of fluid from nozzle. This phenomenon is well described in Figure 4c. Finally, Figure 4d represents the volumetric flow of working fluid through the nozzle towards the substrate. Two trends in each graph occur due to 2 Hz frequency selection for the simulation.

**Figure 5:**
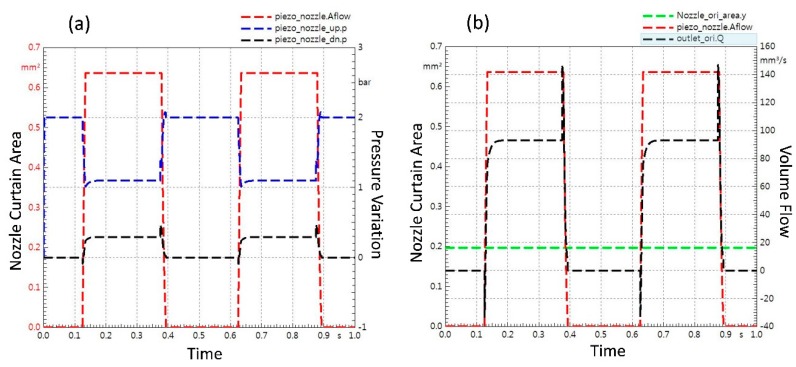
Nozzle opening area variation with tappet movement: (**a**) pressure variation in fluid chamber inside nozzle due to change in nozzle opening area; (**b**) influence of nozzle curtain area on fluid volume flow through nozzle.

Curtain area of nozzle changes with movement of nozzle. With the up and down movement of needle, pressure variation inside the fluid chamber of the nozzle is plotted in Figure 5a. Similarly, Figure 5b visualizes the fluid volume flow through nozzle with respect to curtain area of nozzle in detail while keeping the cross-sectional area of nozzle constant.

**Figure 6:**
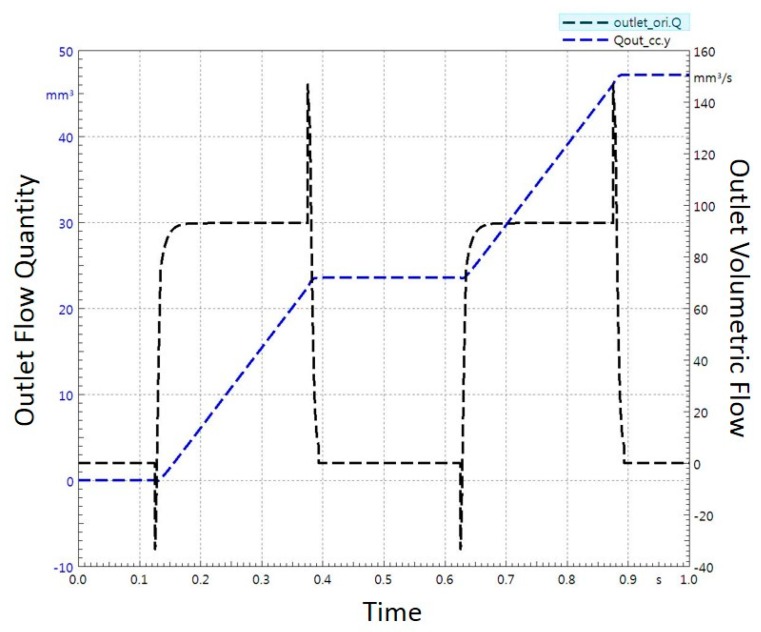
Plot of fluid volumetric flow rate and dispensed droplet volume with respect to open time.

The liquid flow rate out of nozzle in addition with volume flow quantity is presented in Figure 6. The simulation shows that the dispenser would generate about 23.60 μL volume per dispensed droplet for 260 ms of open time.

## 4. Experimental Results and Analysis

### 4.1. Laser Displacement Sensing Experiment

**Figure 7:**
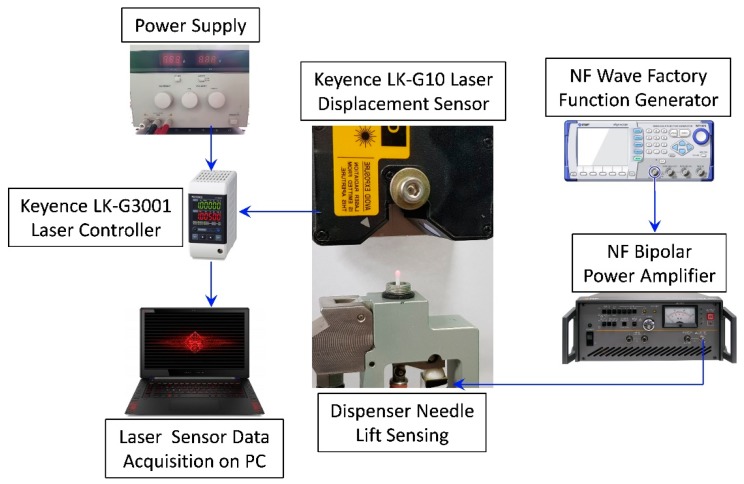
The experimental configuration for needle displacement measurement of jet dispenser.

In order to observe the proper workability of the hinge-lever type dispenser prototype, an experimental setup is devised to observe the amplification due to the class-one lever that is adopted in this dispenser. Before attaching nozzle and proceeding with jetting experiments, bare needle of the jet dispenser is assembled with all necessary components of dispenser. The piezoelectric stacked actuator is connected to the 30 MHz multifunction generator (NF Wave Factory, Yokohama, Japan) by connecting it to the bipolar voltage amplifier by NF corporation [47]. The tappet motion of dispenser is observed by square waveform at 50 percent duty setting with the help of very sensitive laser displacement sensor (LK-G15 by KEYENCE, Itasca, IL, USA) [48]. The reference distance from laser is kept under 10 mm and instructions on laser sensor catalogue were properly followed. The KEYENCE laser sensor was attached to the DC power supply and the sensor was controlled by the Laser controller module and LK Navigator program that was installed in laptop for data collection. The displacement of the tappet was measured using a square waveform within the working limit of laser sensor range. The needle of the jet dispenser was pointed toward the laser sensor and piezostack was excited by function generator and voltage amplifier. The readings, sensed by the laser sensor, were recorded and saved in the laptop for further processing. From laser sensor, 8000 sample points were collected and the graph was plotted against the tappet displacement sensed by the laser. The overall experimental setup for tappet movement measurement is shown below in Figure 7.

**Figure 8:**
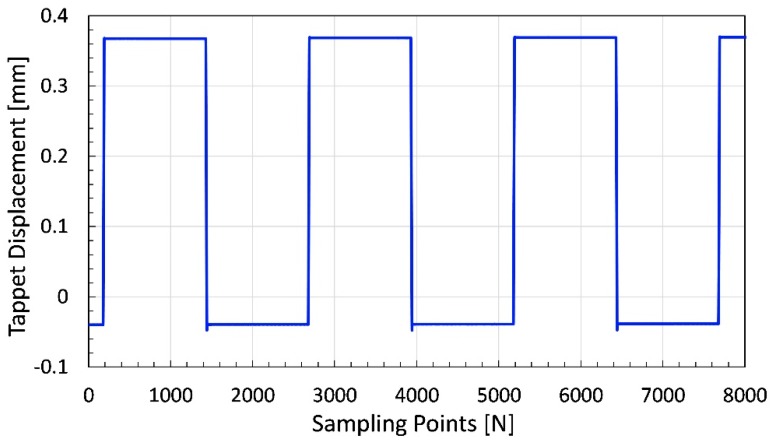
Tappet movement and displacement measurement result.

The feed from the laser sensor represents the needle displacement of jet dispenser as shown in Figure 8. The minimum displacement recorded by sensor is −0.0478 mm whereas the maximum displacement comes out to be 0.3766 mm. Thus, the experimentally measured overall displacement of needle is 0.4244 mm or 424.4 μm as tabulated in Table 3.

The theoretical value for amplified displacement is 380 μm but if we add the input displacement of 60 μm as well, the value becomes 440 μm. In summary, the measured the amplified displacement of the needle is 15.6 μm lesser than the predicted and theoretically calculated value. One of the reasons behind the reduction in amplified displacement can be misalignment of the needle or piezostack. However, the tappet displacement of more than 420 μm is well suited for multiple dispensing applications [20,21,49].

### 4.2. Fluid Dispensing Experimentation 

**Figure 9:**
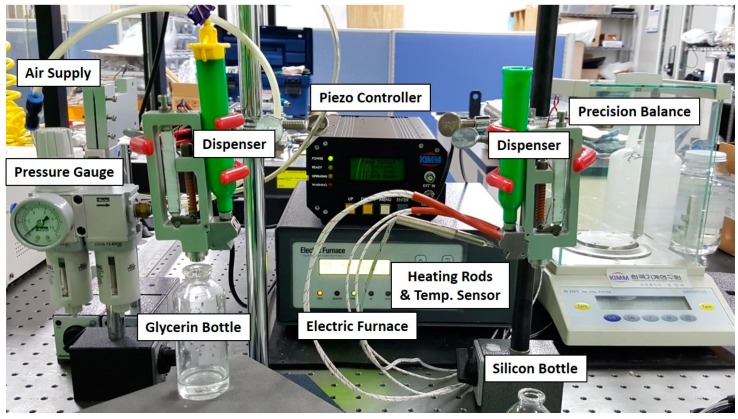
Jetting experiment assembly and test system for hinge-lever type piezo-driven dispenser.

The jet dispenser components are assembled together for experimental evaluation of the working prototype of fabricated jet dispenser. The experiments are divided into two categories. In the first case, the parameters considered in the simulation are followed to observe the comparative jetting with respect to simulation. Afterward, detailed experimentation is carried out using liquid glycerin and high viscosity liquid silicone. Furthermore, influence of crucial parameters like frequency, nozzle diameter, and open time on dispensed mass and dispensed volume is evaluated experimentally. The piezoelectric driven dispenser experimental setup can be seen in Figure 9.

The displacement measurement experiment of a dispenser, as mentioned in the previous section, is carried out using function generator for controlling the waveform and response of the piezoelectric stacked actuator. However, the function generator does not provide proper control over the excitation of the piezoelectric actuator. Thus, a piezo-controller was fabricated to drive the piezoelectric actuator. The design and fabrication process of piezo-controller being used to control the piezostack excitation is beyond the scope of this paper, hence the specifications and control range of piezo controller is tabulated in Table 4 for reference. Tappet lift is controlled using following parameters with respect to Figure 2d.

#### 4.2.1. Dispensing of Glycerin 

**Figure 10:**
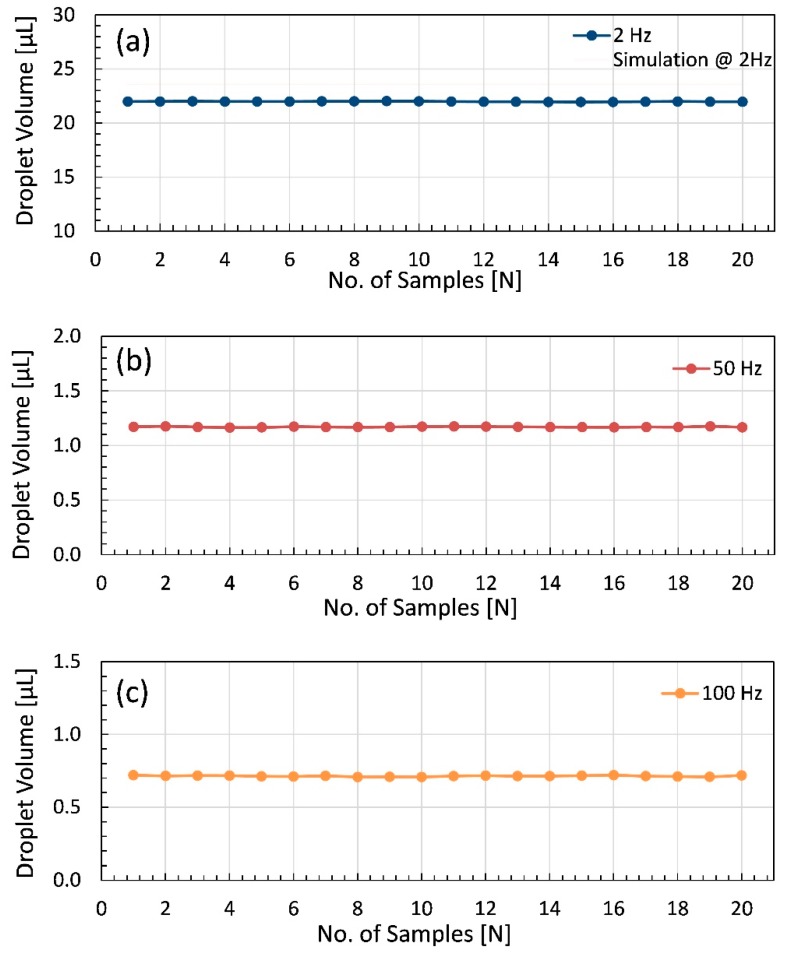
Volume of dispensed droplets of glycerin at various frequencies: (**a**) droplet volume at 2 Hz frequency and 250 ms open time with respect to simulation result at 2 Hz frequency and 260 ms open time; (**b**) droplet volume at 50 Hz frequency and 10 ms open time; (**c**) droplet volume at 100 Hz frequency and 5 ms open time.

This part elaborates the experiments carried out to observe the dispensing of 99.50% pure glycerin with respect to variation in controlling parameters like size of outlet nozzle and open time of dispenser. In addition, the experimental parameters were selected to observe the jetting of the fabricated dispenser in relation to simulation result for the validation of simulation. The reason behind the selection of glycerin with 99.50 % purity is that its viscosity at 20 °C is 1337 mPa·s, which is closely related to the viscosity of the hydraulic fluid (1328 mPa·s) used in simulation environment [33,50,51]. The experimental setup for glycerin experiment is shown on the left side of Figure 9, which comprises of a needle-type proposed dispenser, pressurized air supply line mounted on glycerin syringe with a pressure gauge and regulator to control the supply pressure of air. Pressurized air at 2 bar is supplied throughout the experiment for glycerin. The piezo stack is controlled by piezo-controller, and mass of the dispensed droplets ejected through a nozzle of 300 μm diameter is measured by precision balance and then volume of the dispensed drop is calculated using density of glycerin that is 1.26 g/cm^3^ and the experiment is carried out at 20 °C temperature. In order to report the data accurately, 20 repetitions for each reading are carried out and the averaged values are considered as the effective volume of the glycerin. In addition, at 2 Hz, 50 Hz, and 100 Hz frequency, the collected data for 20 consecutive readings is plotted to observe the influence of frequency variation and to report the data with minimum error as presented in Figure 10.

At first, the piezo controller is set at 2 Hz frequency, and the open time is kept at 250 ms. The dispensed volume per dispensed droplet is obtained and plotted in Figure 10a. The average volume of a single drop is equal to 22 μL whereas the standard deviation is as minimal as 0.03 μL. The simulation of dispensing is also carried out at similar settings by keeping the frequency at 2 Hz but the open time calculated by interpolation of simulation results shows the open time of 260 ms on the simulated model. The dispensed volume of 23.60 μL per single drop is obtained through simulation. The reason behind the difference of 1.6 μL volume in simulated and experimental results lies in the fact that the simulation model calculated the dispensing for 260 ms whereas during experiments open time of 250 ms is applied as mentioned earlier. Thus, the additional 10 ms of open time results in an extra 1.6 μL of dispensed liquid per droplet in simulation. The simulation was carried out prior to the fabrication of dispenser, only using detailed geometry from its drawing and model. Thus, only the simplistic model of simulation is considered for validation through experimentation. After fabrication of dispenser, preliminary attention is focused on experiments for the exploration of the newly fabricated dispenser. Thus, similar procedure is adopted for the second and third case, where the frequency is kept at 50 Hz and 100 Hz with 10 ms and 5 ms open time, resulting in 1.2 μL and 0.7 μL of average dispensed droplet volume with standard deviations of 0.003 μL and 0.004 μL as shown in Figure 10b,c, respectively. 

**Figure 11:**
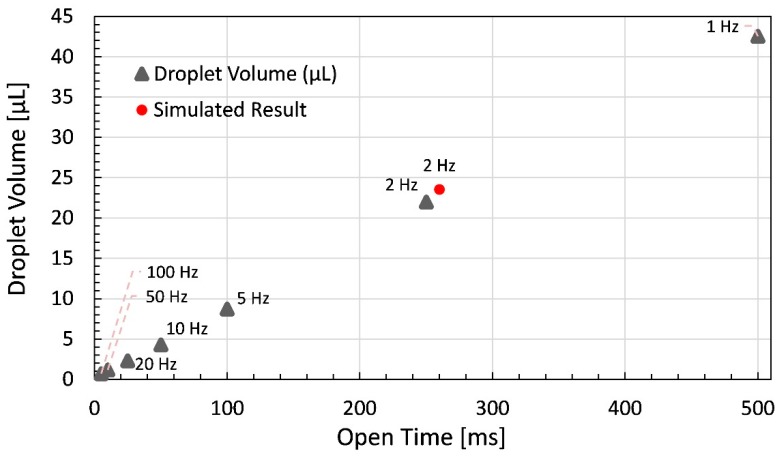
Droplet volume of dispensed droplets plotted against open time with respect to respective frequency in addition to simulation result at 2 Hz frequency and 260 ms open time.

Multiple readings for dispensing with each setting are taken to obtain the data to be presented in a wide spectrum to represent the workability of proposed dispenser. In Figure 11, the droplet volume of dispenser is plotted against open time while varying the frequency as well. 

For dispenser settings, the working constraint is that the open time of dispenser can never be greater than 10 ms, as a period of 10 ms is equal to 100 Hz. Thus, for higher open time the frequency was also reduced and vice versa. For the case shown below, a nozzle of 300 μm diameter is used with glycerin as working fluid at 20 °C. The open time variation can be seen on x-axis of graph which is varied as 5 ms, 10 ms, 25 ms, 50 ms, 100 ms, 250 ms, and 500 ms with the frequency variation of 100 Hz, 50 Hz, 20 Hz, 10 Hz, 5 Hz, 2 Hz, and 1 Hz, respectively. The interesting results are achieved through simulation at 2 Hz frequency with 260 ms of open time which is shown in the graph by a red dot and the reason behind variation of 1.6 μL of drop volume has been explained previously.

**Figure 12:**
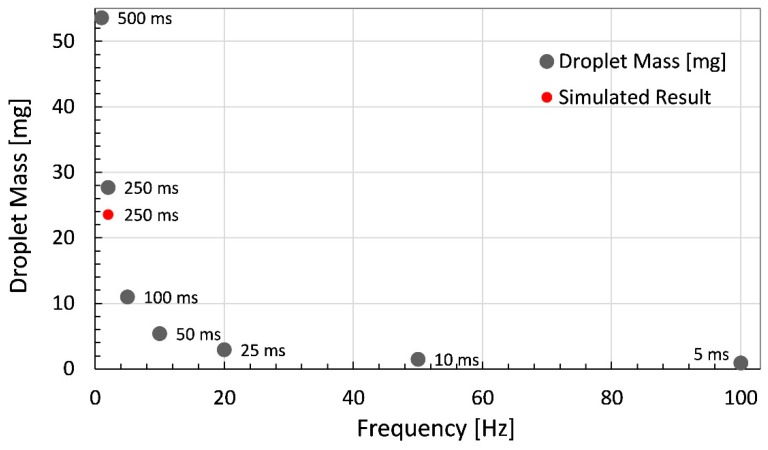
Droplet mass of dispensed droplets plotted against the frequency with respect to appropriate open time in addition to simulation result at 2 Hz frequency and 260 ms open time.

Similarly, the dispensed mass of droplets using 99.50% pure glycerin is plotted with respect to variation in dispenser frequency while keeping the value of open time appropriate for selected frequency. The minor difference of 2.0 mg in simulated result from experimental one is due to 10 ms extra open time difference with respect to experiment in the modelling as shown in Figure 12. Thus, the experiments using glycerin provide positive feedback for extension of experiments using high viscosity liquid silicone, as the proposed piezoelectrically driven dispenser exhibited reliable trends with respect to the simulation model in terms of open time and frequency variation. The effect of variation in dispensed droplets with respect to pressure variation has already been reported in literature for various kinds of dispensers employing displacement amplification mechanism for piezostacks [9,33,34]. 

The section about dispensing of glycerin provides details of experimental assembly and experiment arrangements in addition to basic workability of dispenser based on the averaged volume of multiple dispensing readings for each set of frequency and open time. Only one nozzle size was considered in this section to observe the dispensing of the prototype with reference to simulation parameters. The simulation and experimental results match well with minimal differences. Thus, more viscous liquid is considered for dispensing using the proposed prototype and the nozzle size is also varied in addition to variation in open time and frequency.

#### 4.2.2. Dispensing of Liquid Silicone

The four sizes of carbide metal nozzle (by VERMES Microdispensing GmbH, Otterfing, Germany) including 800 μm, 500 μm, 300 μm, and 100 μm diameter with the circumference and height of 4 mm and 1.75 mm for each, are used in this experiment, respectively [46]. In addition, liquid silicone of 12,500 mPa·s viscosity (No. 12500 cps by BROOKFIELD Ametek, Middleboro, MA, USA) is adopted as dispensing liquid with pre-heating at 50 °C by electric furnace as shown on right side of the experimental set up in Figure 9 [52]. As the liquid silicone being used has about 9.4 times higher viscosity than glycerin at 20 °C temperature, so heating was carried out in addition with increase in air supply pressure from 2 bar to 5 bar for continuous flow of fluid through fluid channel and cavity for the sake of bubble-free and non-disruptive continuous supply to the nozzle. The Brookfield liquid silicone being used changes viscosity in a pre-determined manner, thus becoming less viscous (6250 mPa·s) at elevated temperature (50 °C). 

**Figure 13:**
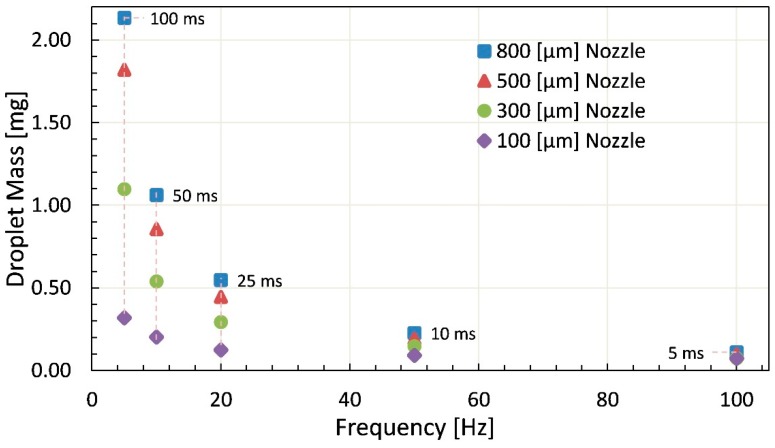
Droplet mass of dispensed droplets using 12,480 mPa·s liquid silicone by Brookfield plotted against frequency with respect to varying open time and nozzle diameter.

The experiment is carried out using each nozzle for variety in open time and frequency. For liquid silicone experiments, frequency is controlled between 5 Hz to 100 Hz whereas the open time selection range is kept from 5 ms to 100 ms. The idea is to consider the proposed dispenser for application of epoxy and bonding materials with higher viscosities that do not need very high-frequency operation. The multiple measurements were averaged to plot each point with respect to specified settings in terms of the dispensed mass of jetted droplet with respect to variation in frequency, open time and nozzle size as shown in Figure 13. Each point on the graph has four specifications mentioned on graph to provide insight into the wide range of working capability of the designed dispenser. The graph represents a decreasing trend in droplet size from left to right in terms of specified open time. In addition, the influence of nozzle diameter on jetting is depicted in graph that elaborates the reduction in droplet mass from the top toward bottom, and vice versa.

**Figure 14:**
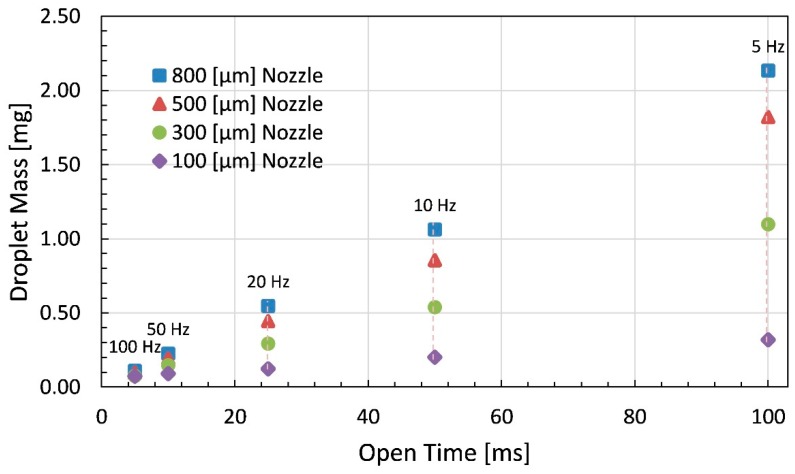
Droplet mass of dispensed droplets using 12,480 mPa·s liquid silicone by Brookfield plotted against open time with respect to varying frequency and nozzle diameter.

Finally, the experimental data are summarized in terms of open time on the x-axis whereas pointing out the influence of nozzle diameter and frequency on the droplet mass as can be seen in Figure 14.

The open time is directly proportional to the drop size, whereas frequency is inversely related to it. With the increase in nozzle size the mass of dispensed droplet increases whereby reduction in nozzle size results in fine or ultra-fine droplets. The increase in viscosity of liquid reduces the dispensed droplet mass and it can be compensated by employing big nozzle diameter in addition to higher applied air pressure. Thus, the parameters like open time and frequency, in addition to the pressure of supplied air and tappet lift as well as the viscosity of liquid are the most crucial parameters for the dispenser under consideration. 

## 5. Discussion

On the basis of geometry-based simulation of class-one lever, working prototypes of piezo-driven hinge-lever type liquid jet dispenser is fabricated and experimentally evaluated for its dispensing capabilities up to 100 Hz frequency with minimal open time of 5 ms by the help of a piezo controller. The nozzles from 100 μm diameter to 800 μm are employed to observe their influence on the dispensing of droplets. In addition, jetting of two liquids of 1328 mPa·s and 12,500 mPa·s is successfully demonstrated by the proposed model of jet dispenser at two air supply pressure values. 

Most of the dispensers currently in use either use two piezoelectric actuators or utilize very famous and commonly adopted rhombus-type displacement amplification-mechanism. The former mechanism is not economical as it uses two piezostacks whereas the latter mechanism may cause some issues due to structural deformation of metal-frame on which it relies completely. In this research, the classic approach is adopted and the need of second piezostack or metal structure for jetting is met by using a spring with high spring constant that provides a simple solution to a complex problem and can be replaced in no time. 

The highest achieved droplet mass for glycerin is 53.6 mg at 1 Hz frequency and 500 ms open time with 2 bar supply pressure whereas lowest droplet mass for glycerin comes out to be 0.9 mg at 100 Hz frequency and 5 ms of open time while the other parameters are kept constant. The piezo-driven dispenser can adopt a heater as well for pre-heating of high viscosity liquids for smooth flow of fluid through the fluid channel. Using 12,500 mPa·s high viscosity liquid silicone minimum droplet mass of 0.07 mg at 100 Hz frequency and 5 ms open time with 100 μm nozzle, whereas maximum droplet mass of 2.13 mg at 5 Hz frequency and 100 ms open time with 800 μm nozzle is achieved through this dispenser.

The results provide overall preliminary safe working range of designed dispenser. Further experiments will be carried out to obtain droplet volume in the nano-liter range by further reducing the open time to 1 ms or lower if possible, using a piezo-controller to test the extreme limits of the dispenser. In addition, calibration for specified volume delivery will be carried out with overall performance evaluation of working limits of dispenser for future automatic control of dispenser.

## 6. Conclusions

A compact and simplistic model of piezo-driven, hinge-lever type, liquid dispenser is simulated and the parameters are used for accurate fabrication of working prototype of a normally-closed dispenser. Design and development procedure of hinge-lever type dispenser is elaborated and the preliminary experimentation is carried out to observe the influence of various operating parameters on the performance of the piezo-driven jet dispenser and their effect on the jetting with experimental validation is reported in this work. For minimization of error, every single entry in the experimental set of data was measured 20 times and standard deviations were calculated analytically. It is confirmed that the reduction in open time and increase in frequency results in minimum average mass of droplet. Finally, dispenser workability for different viscosity liquids is addressed and useful experimental results are obtained for 1 Hz to 100 Hz of frequency and 500 ms to 5 ms of open time, respectively, that can be used as a reference for future calibration and performance improvement of the proposed dispenser. 

The results presented in this research clearly witness the dependence of dispensed droplet size over the nozzle diameter including other parameters. This research provides a blue-print for various parameter settings to obtain desired droplet size. However, further experiments will be carried out to observe the influence of variation in needle lift, supply pressure, and other controlling parameters on the meniscus, droplet shape, and droplet volume to clearly calibrate the newly designed dispenser for applications involving automatic control of dispenser in the future.

## Figures and Tables

**Table 1:** Lever length, amplification ratio, and output force calculation.

c_1_ (mm)	c_2_ (mm)	R = c_1_/c_2_	a_1_ (μm)	a_2_ (μm)	F_1_ (N)	F_2_ (N)
1.5	9.5	6.34	60.0	380.0	3500.0	552.6

**Table 2:** Parameters and their values used in the simulation.

Parameter	Value
**Density of Fluid**	0.8916 g/cm^3^
**Dynamic Viscosity**	1328.0 mPa·s
**Temperature**	0 °C
**Nozzle Diameter**	300 μm
**Feed Pressure**	0.2 MPa
**Amplification Ratio**	6.34
**Piezo Stroke**	55 μm
**Spring Stiffness**	25.3 N/mm

**Table 3:** Laser displacement-sensor experiment results.

Maximum Displacement	Minimum Displacement	Overall Displacement	Overall Displacement	Sampling Points
−0.0478 mm	0.3766 mm	0.4244 mm	424.4 μm	0–8000 N

**Table 4:** Piezo controller settings applied for piezostack excitation.

Rising Time (RT)	Open Time (OT)	Falling Time (FT)	Frequency Range	Voltage
0.5 ms	5–500 ms	0.1 ms	1–100 Hz	130 V

## Supplementary Materials

The following are available online at https://www.mdpi.com/2072-666X/11/2/117/s1, Video S1: 1 Hz Dispensing.
